# Preoperative chemosensitivity testing as Predictor of Treatment benefit in Adjuvant stage III colon cancer (PePiTA): Protocol of a prospective BGDO (Belgian Group for Digestive Oncology) multicentric study

**DOI:** 10.1186/1471-2407-13-190

**Published:** 2013-04-12

**Authors:** Alain Hendlisz, Vassilis Golfinopoulos, Amelie Deleporte, Marianne Paesmans, Hazem El Mansy, Camilo Garcia, Marc Peeters, Lieven Annemans, Caroline Vandeputte, Marion Maetens, Ivan Borbath, Damien Dresse, Ghislain Houbiers, Michael Fried, Ahmad Awada, Martine Piccart, Jean-Luc Van Laethem, Patrick Flamen

**Affiliations:** 1 Medicine Department, Institut Jules Bordet, Université Libre de Bruxelles, Brussels, Belgium; 2 Data Center, Institut Jules Bordet, Université Libre de Bruxelles, Brussels, Belgium; 3 Nuclear Medicine Department, Institut Jules Bordet, Université Libre de Bruxelles, Brussels, Belgium; 4 Department of Oncology, Antwerp University Hospital, Antwerp, Belgium; 5 Health Economics, Department of Public Health, Ghent University, Ghent, Belgium; 6 Gastroenterology Department, Cliniques Universitaires Saint-Luc, Université Catholique de Louvain, Brussels, Belgium; 7 Service de Chirurgie Abdominale et Générale, Centre Hospitalier Régional La Citadelle, Liège, Belgium; 8 Service d’Oncologie et d’Hématologie, CH Saint Joseph, Liège, Belgium; 9 Oncology department, Ziekenhuis Netwerk Antwerpen, Antwerpen, Belgium; 10 Gastroenterology Department, Erasme Hospital, Université Libre de Bruxelles, Brussels, Belgium

**Keywords:** Colon cancer, Adjuvant, Early assessment, Chemosensitivity, FDG-PET, PET/CT

## Abstract

**Background:**

Surgery is a curative treatment for patients with locally advanced colon cancer, but recurrences are frequent for those with stage III disease. FOLFOX adjuvant chemotherapy has been shown to improve recurrence-free survival and overall survival by more than 20% and is nowadays considered a standard of care. However, the vast majority of patients will not benefit from receiving cytotoxic drugs because they have either already been cured by surgery or because their tumor cells are resistant to the chemotherapy, for which predictive factors are still not available.

Identifying which patients are unlikely to respond to adjuvant chemotherapy from among those who are eligible for such treatment would be a major step towards treatment personalization. It would spare such patients from unnecessary toxicities and would improve the allocation of societal healthcare resources.

**Methods/design:**

PePiTA is a prospective, multicenter, non-randomised trial built on the hypothesis that preoperative chemosensitivity testing using FDG-PET/CT before and after one course of FOLFOX can identify the patients who are unlikely to benefit from 6 months of adjuvant FOLFOX treatment for stage III colon cancer.

The study’s primary objective is to examine the ability of PET/CT-assessed tumor FDG uptake after one course of preoperative chemotherapy to predict the outcome of adjuvant therapy, as measured by 3-year disease-free survival.

Secondary objectives are to examine the predictive value of changes in PET/CT-assessed tumor FDG uptake on overall survival, to define the best cut-off value of FDG uptake for predicting treatment outcome, and to analyse the cost-effectiveness of such preoperative chemo-sensitivity testing.

At study planning, exploratory translational research objectives were 1) to assess the predictive value of circulating tumor cells for disease-free survival, 2) to examine the predictive value of single nucleotide polymorphisms for disease-free survival with respect to genes related either to toxicity or to drug targets, 3) to assess genomic rearrangements associated with response or resistance to FOLFOX treatment, 4) to identify an immunologic signature associated with metabolic tumor response to FOLFOX therapy and, finally, 5) to create a bank of frozen tumor samples for future studies.

**Discussion:**

PePiTA is the first study to use the primitive tumor chemosensitivity assessed by metabolic imaging as a guidance for adjuvant therapy in colon cancer. It could pave the way for tailoring the treatment and avoiding useless toxicities for the patients and inadequate expenses for the society. It could also give an interesting insight into tumoral heterogeneity, resistance to chemotherapy, genetic predisposants to oxaliplatin toxicity and immune response to cancer.

**EudraCT number:**

2009-011445-13

**Trial registration:**

ClinicalTrials.gov number, NCT00994864

## Background

### Adjuvant treatment of colon cancer

Colon cancer is a major public health problem accounting for over one million new cases per year worldwide [1]. Surgery is the only curative therapy available for localised colon cancer; however, the disease is associated with a significant recurrence rate for which the depth of tumor penetration within the intestinal wall and the presence of involved lymph nodes, as described in the TNM classification system [2], are major prognostic factors.

In stage III colon cancer, postoperative adjuvant chemotherapy is associated with a statistically significant improvement in disease-free survival (DFS) and overall survival (OS) compared to surgery alone. The National Surgical Adjuvant Breast and Bowel Project (NSABP) C-01 trial, reported in 1988, was the first study to show such a survival advantage [3]. Drug regimens were improved substantially over the years and a 6-month combination of 5 flurouracil (5FU), folinic acid, and oxaliplatin (FOLFOX) became the reference treatment in 2004 showing a 0.76 hazard ratio (HR) (95% confidence interval [CI] 0.62–0.92) for relapse favouring FOLFOX compared to fluorouracil plus folinic acid [4]. In a population of stage II-III colon cancer, FOLFOX is associated with a 3-year 72.2% DFS and a 78.5% 5-year survival rate. The current consensus is that adjuvant chemotherapy is indicated for stage III colon cancer. However, because of a lower magnitude of benefit, adjuvant chemotherapy remains controversial for stage II colon cancer, especially for low-risk patients [5].

Acute toxicity to the FOLFOX regimen in the adjuvant setting has been well described as moderate to severe [4], but very little is known with respect to long-term side effects. With delayed cardiac [6], cognitive [7,8] and oncogenic [9] side effects increasingly being reported in cancer survivors treated with adjuvant therapies, long-term side effects are a major concern associated with every treatment given with curative intent.

It is challenging to identify which patients are unlikely to benefit from adjuvant therapy, because no measurement is possible once a tumor has been removed. Being able to predict this successfully would bring great benefit, because this would potentially spare many patients from acute and delayed drug-induced toxicities, and avoid the useless spending of public and private healthcare resources.

Standard response measurements (WHO [10], RECIST [11], and modified RECIST [12]) rely entirely upon measuring the size of the tumor with computerized tomography scan (CT scan), ultrasound or magnetic resonance imaging (MRI) and only apply under restrictive conditions (well defined lesions, adequate minimum size, at least six weeks of chemotherapy). In the absence of any reliable chemosensitivity predictors, using such measurements to determine response means giving potentially ineffective treatments to patients for long periods of time.

However, early response detection techniques are now emerging, of which ^18^ F Fluorodeoxyglucose (FDG) positron emission tomography (PET)/CT and circulating tumor cells (CTCs) are the most promising.

### Positron emission tomography

The most frequently used tracer for PET/CT in the clinical setting is FDG, a marker of glycolysis, which is generally increased in malignant tumors. The uptake of FDG is related to the viable tumor cell load, and indirectly to the proliferative activity. PET/CT imaging has been able to measure a decrease in FDG uptake very early after exposure to treatment in various settings. In the context of neoadjuvant treatment of breast cancer, the reduction of FDG uptake measured at two weeks after the first dose of chemotherapy has been shown to predict the late FDG-PET/CT response measured four weeks after the end of chemotherapy, and to correlate with histopathological response [13].

A correlation between an early metabolic response after one or two courses of therapy and patient outcome has also been found in diffuse large cell lymphoma and Hodgkin’s disease [14], high-burden follicular lymphoma [15], lung cancer [16,17], and locally advanced oesophagogastric junction tumors treated with neoadjuvant chemotherapy [18]. In the latter indication, metabolic imaging-based assessment of response has already been used to develop trial designs aimed to tailor treatments according to the level of efficacy [19,20].

In advanced colorectal cancer, reports on the correlation of patient outcomes with FDG-PET/CT-based metabolic response assessment after 1 or 2 months of chemotherapy are inconsistent [21-23], probably partially because of methodological issues in multi-metastatic assessment. Early assessment after one course of therapy seems to be a more promising approach in light of recent findings that show excellent negative predictive value in the absence of morphological response and good correlation with the overall patient survival [24].

In locally advanced rectal cancer, several independent research groups have confirmed the high negative predictive value of metabolic assessment, demonstrating that the FDG uptake remains unchanged if the tumor is not sensitive to radio-chemotherapy [25,26].

### Circulating tumor cells

CTCs can be detected in the peripheral blood of patients with advanced tumors and could be useful to monitor treatment efficacy in metastatic colorectal carcinoma (CRC) [27-30] and breast cancer [31-33]. A recent report by Uen et al. suggests an association between the persistence of CTCs after curative surgery for CRC and recurrence risk [34]. No data exist concerning their use in the neoadjuvant setting to monitor therapeutic response. To assess the potential role of CTCs in locally advanced colon cancer, we designed a substudy in the PePiTA trial to analyze the prognostic value of baseline CTCs and the predictive value of CTC count modifications after one course of preoperative chemotherapy for adjuvant therapy outcome.

### Single nucleotide polymorphisms

Predicting the toxicity of anti-cancer drugs is complex. DNA changes (mutations, polymorphisms and gene hypermethylation) are increasingly implicated in the observed variation of toxicity among patients. Knowledge of these characteristics would allow treatments to be tailored according to individual susceptibility. This point will be addressed prospectively by performing single nucleotide polymorphism (SNP) testing before the preoperative course of chemotherapy.

### Genomic rearrangements

Recent advances in next generation sequencing (NGS) technologies and data analysis have enabled us to explore specific somatic rearrangements and aberrations in the cancer genomes of individual patients in a cost effective and time efficient manner [35]. Tumor cells releasing naked DNA into blood after apoptosis or necrosis provide hope for a new approach to cancer detection through the quantification and analysis of circulating free DNA. The idea of evaluating blood samples for mutant DNA is particularly attractive, not only because it could be used to detect several cancer types, but also because blood samples can easily be collected during patient follow-up as so-called “liquid biopsy” [36]. If the NGS analysis in this study demonstrates that the detection and quantification of specific tumoral genomic rearrangements in circulating DNA provides a sensitive and specific measure of disease progression, treatment response and prognosis in patients with colon cancer, this will have a major impact on the future clinical treatment of the disease.

### Tumor immune infiltration

At present, there are no clinical data available reporting the immune response (reflected by the type, density and function of tumor infiltrating lymphocytes (TILs)) of colon cancer after preoperative chemotherapy. Chemo- and targeted therapies that potentially stimulate the immune system [37] or inhibit Treg [38] could modify the immune infiltration of primary or metastatic tumors. In this regard, preclinical data (CRC cell lines) suggest that oxaliplatin induces immunogenic death of CRC cells, and that this effect determines its therapeutic efficacy in patients with CRC [39]. Therefore, in this study, particular attention will be given to the analysis of TILs in tumors resected after chemotherapy.

### Study hypothesis

PePiTA is built on the hypothesis that testing FDG-PET/CT metabolic changes before and after one course of FOLFOX given preoperatively will identify the patients with stage III colon cancer who are most unlikely to have significant benefit from adjuvant treatment using the same chemotherapy regimen after surgery for 6 months.

## Methods

### Study design

**Figure 1:**
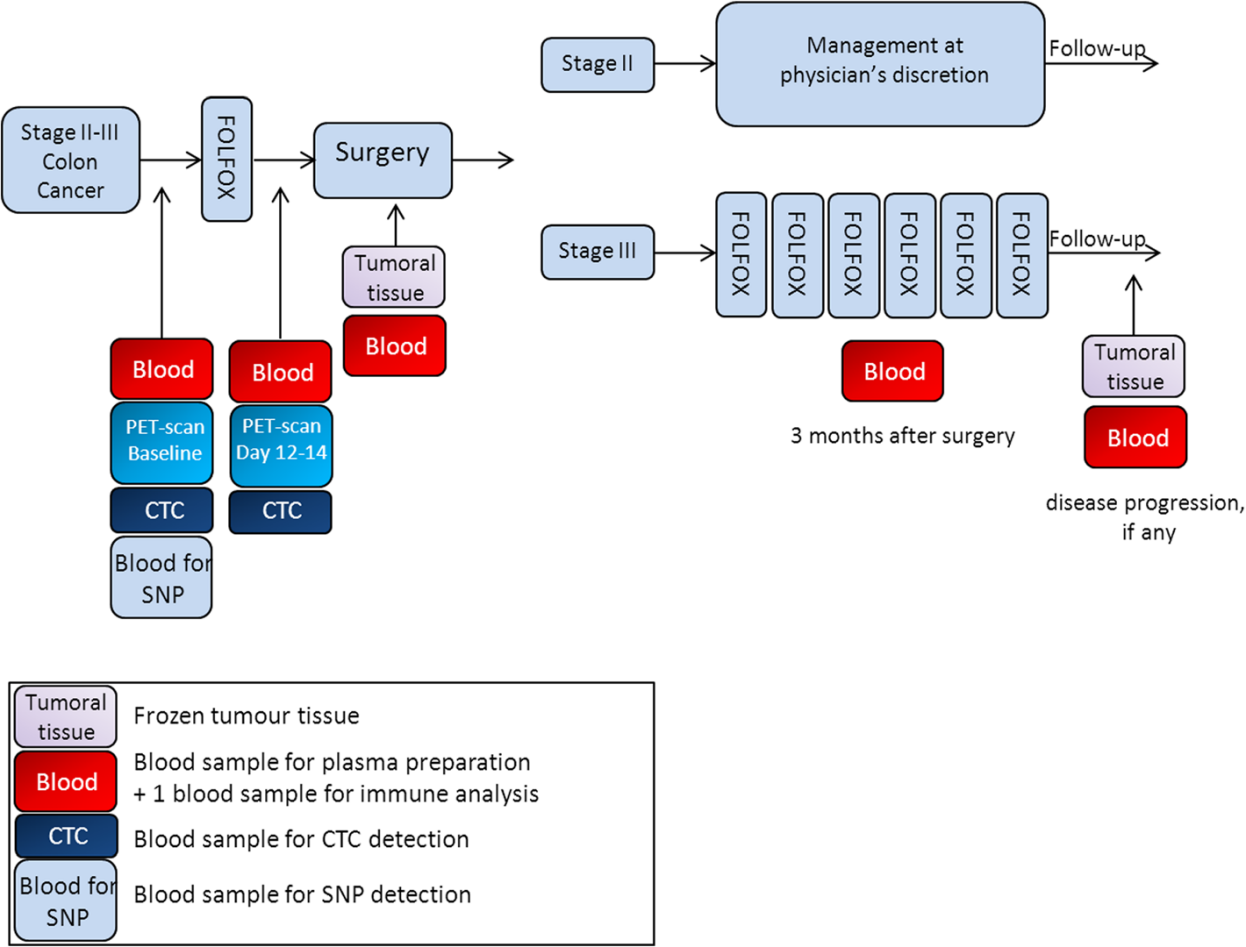
Study design.

PePiTA is a prospective, multicenter, non-randomised trial. Patients with histologically confirmed colon adenocarcinoma compatible with clinical stage III are eligible for study screening. The study design is illustrated in Figure 1.

### Objectives

PePiTA’s primary objective is to examine the ability of PET/CT-assessed tumor FDG uptake after one course of preoperative chemotherapy to predict the outcome of adjuvant therapy, as measured by 3-year DFS among patients with pathological stage III.

Secondary objectives are to examine the predictive value of changes in PET/CT-assessed tumor FDG uptake on OS, to define the best cut-off value of FDG uptake for predicting treatment outcome, and to analyse the cost-effectiveness of such preoperative chemosensitivity testing.

Exploratory (translational research) objectives in the context of several sub-studies are 1) to assess the predictive value of CTCs for DFS, 2) to examine the predictive value of SNPs for DFS with respect to genes related to toxicity or drug targets, 3) to assess genomic rearrangements associated with response or resistance to FOLFOX treatment, 4) to identify an immunologic signature associated with metabolic tumor response to FOLFOX therapy and, finally, 5) to create a bank of frozen tumor and blood samples for future translational research studies.

### Patient selection criteria

### Inclusion criteria

Patients 18 years or older, diagnosed with adenocarcinoma of the colon compatible at endoscopy examination with a stage III cancer (invasion of more than one third of the circumferential colonic lumen) and considered curatively resectable (R0) based on standard preoperative evaluations, are eligible for inclusion.

The patients must have good performance status (WHO performance status 0 or 1), never have received chemotherapy or pelvic irradiation, have signed informed consent prior to any study specific procedures, and have agreed to use effective contraception during the study and until six months thereafter.

### Exclusion criteria

In addition to pregnant or breast-feeding women, excluded from the study are patients identified with any of the following conditions or characteristics: suspected metastatic disease; rectal cancer (located within 15 cm from the anal verge by endoscopy or under the peritoneal reflection at surgery); serious illness like inflammatory bowel disease, central nervous system disease, peripheral neuropathy, clinically relevant coronary artery disease, history of myocardial infarction in the last six weeks, high risk of uncontrolled arrhythmia, or previous malignancy in the last five years (except basal-cell carcinoma of the skin or in situ cervical carcinoma); hypersensitivity to any of the components of study treatments; major surgical procedure, open biopsy or significant traumatic injury within 28 days prior to the study; and having incompletely healed wounds or anticipating the need for major surgery during the course of the study.

### Treatment

All eligible patients will undergo FDG-PET/CT imaging at baseline and then after one cycle of FOLFOX [3] preoperative chemotherapy (a 2-hour infusion of 200 mg/m2 leucovorin or equivalent, followed by a bolus of 400 mg/m2 fluorouracil and then a 22-hour infusion of 600 mg/m2 fluorouracil given on 2 consecutive days; a two-hour infusion of 85 mg/m2 oxaliplatin on day 0, simultaneously with leucovorin, using a Y-infusion device) followed by surgery.

After surgery, patients will be fully evaluable in the study and receive adjuvant FOLFOX when they meet the following criteria: the delay between the first PET/CT scan and the start of neoadjuvant FOLFOX was less than 7 days; the second PET/CT scan was performed on D14 (range: D13-D15); postoperative pathology confirms stage III (pTNM) adenocarcinoma of the colon; and CEA level measured one month after surgery is less than 1.5 × ULN.

**Figure 2:**
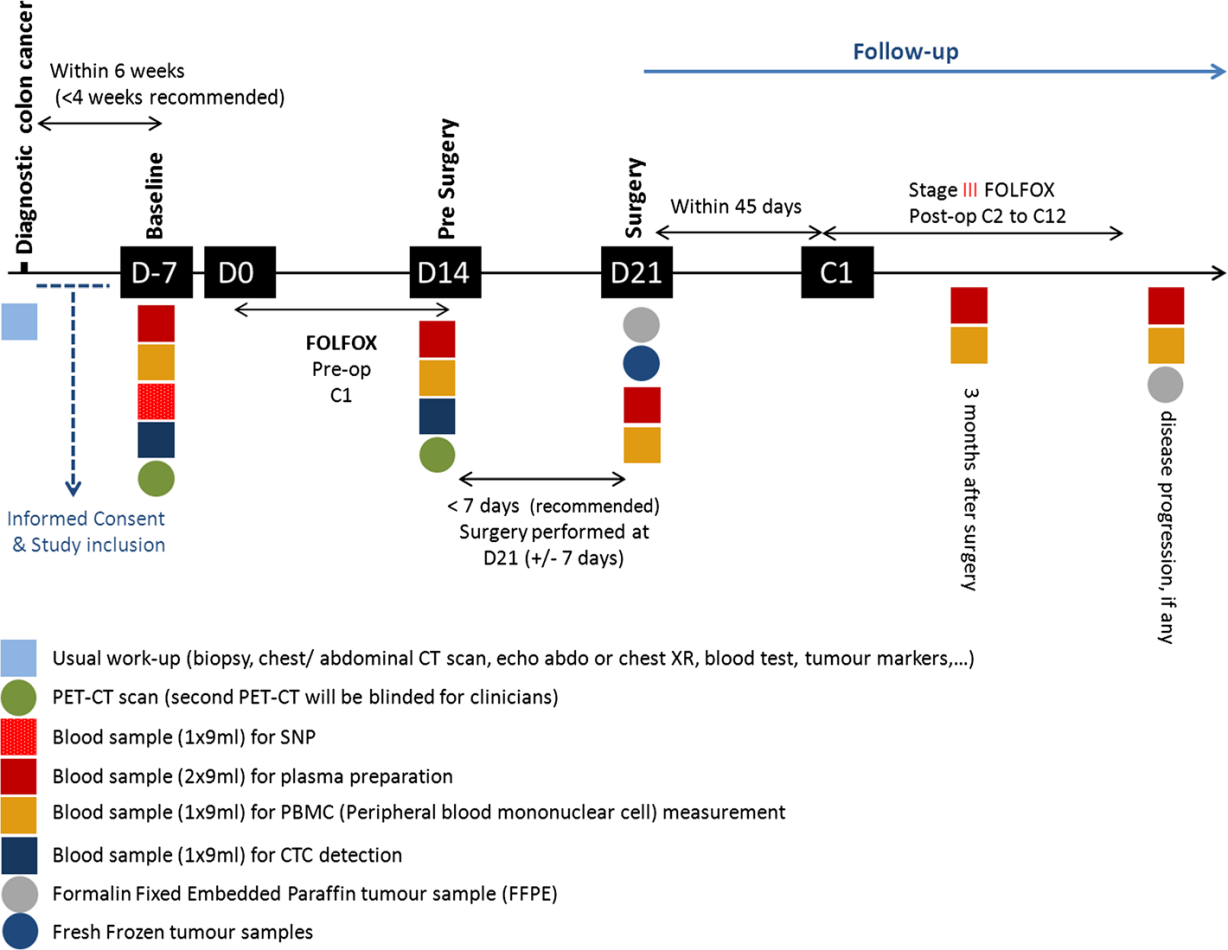
Schedule of assessments.

All patients, both those meeting the inclusion criteria only and those fully evaluable for the primary objective, will be followed for DFS and OS assessment. Time zero for the assessment of time-to-event outcomes is the first day of pre-operative chemotherapy. In case a patient dies or is lost to follow-up before surgery, he or she will be excluded from the analysis of DFS and OS. The schedule of assessments is described in Figure 2.

### FDG-PET/CT imaging

Whole body static FDG-PET/CT scans will be performed as described above. The local clinical investigators will be informed about the results of the baseline FDG-PET/CT. In case this scan shows suspicion of stage IV disease, a targeted confirmatory work-up is required before definite exclusion.

The results of the second FDG-PET/CT will be blinded to all local clinical investigators until there is formal documentation of relapse or progression.

Major efforts are undertaken to standardise patient preparation, FDG-PET/CT acquisition, image reconstruction, and semi-quantification of FDG uptake. This is centrally coordinated by the Imaging Core Lab at Institut Jules Bordet’s Nuclear Medicine Department. Strict standardisation will ultimately allow reliable pooling of the FDG-PET/CT imaging data from all participating centres. All participating FDG-PET/CT centres will be required to adhere to the quality assurance guidelines defined by the Belgian Hospital Physicists Association, and to perform a standard phantom (NEMA-type) acquisition, which will ultimately be used for a cross-calibration of all PET cameras.

The response assessment of the primary tumor will be performed according to the European Organisation for the Research and Treatment of Cancer (EORTC) guidelines: a metabolic response corresponds to a ≥15% decrease of the standardised uptake value max. In case of the presence of locoregional lymph node(s) observed on the baseline FDG-PET/CT, a similar response analysis will be performed for each lesion separately.

### Circulating tumor cells

PePiTA’s CTC substudy aimed to examine the prognostic value of CTCs in colon cancer before and after one course of preoperative chemotherapy and the predictive value of changes in CTCs for adjuvant treatment efficacy after one course of preoperative chemotherapy.

Within 24 hours after collection, 9 ml of peripheral blood per patient have to be sent at room temperature to the laboratory responsible for CTC detection. All experiments and evaluations will require the investigators to be blinded to the clinical status of the patients.

### Single nucleotide polymorphisms

PePiTA’s SNP substudy aims to identify molecular biomarkers to test the value of SNPs 1) to predict chemotherapy toxicity by correlating the biomarkers with clinical toxicity as assessed by NCI-CTCAE v. 3.0, and 2) to predict patient outcome by evaluating genes related to drug targets.

At each follow-up visit, patients are asked to complete a questionnaire about chemotherapy-induced peripheral neuropathy (EORTC QLQ-CIPN20 and Modified Norris scale).

Before preoperative chemotherapy, one EDTA blood sample (VenoSafe EDTA-K_2_) 9 ml has to be obtained and stored at −80°C and then sent to the laboratory responsible for SNP analysis.

Candidates for polymorphism genotyping are:

a. Genes related to drug targets: for the platinum compounds, the DNA repair genes *XRCC1, XRCC3, ERCC1, ERCC2, XPD, hMSH2, MLH1*[40]; for the antimetabolites 5-FU, capecitabine and pemetrexed, *TYMS*[41].

b. Genes related to drug metabolizing enzymes: the *CYP* gene family, coding for cytochrome P450 enzymes, and the *GST* genes, which are linked to drug resistance and toxicity in platinum drugs and 5-FU/platinum combinations [42,43].

### Genomic rearrangements

PePiTA also aims to use NGS technology to identify genomic rearrangements (individual or shared among patients) associated with response or resistance to preoperative chemotherapy guided by FDG-PET/CT metabolic imaging both in tumor tissue and in plasma samples. First, DNA will be extracted from fresh frozen tissue. Subsequently, DNA samples will undergo low-coverage whole-genome sequencing analysis. To follow modifications in tumor-specific rearrangements (as characterized by NGS) in the patient over time, circulating DNA will be extracted from the plasma and patient-specific qPCR protocols will be developed (Figure 2).

### Tumor immune infiltration

The goal of this PePiTA substudy is to identify the immunologic signature associated with metabolic tumor response to preoperative FOLFOX therapy in colon cancer. TILs will be characterized by immunohistochemistry using markers for specific immune cells including cytotoxic T lymphocytes, memory T cells, regulatory T cells, B lymphocytes, and macrophages, amongst others. Immunohistochemical stainings will be performed on each resected formalin fixed paraffin embedded (FFPE) colon cancer tumor, as previously described [44-47]. Next, cDNA microarray analysis (Affymetrix U133 Plus 2.0) and RT-qPCR (Taqman) will be realized on frozen tumors in order to analyze the expression of inflammatory genes, immunosuppressive genes and genes related to the adaptive immune response. Moreover, blood samples will be taken at several time points during patient follow-up (Figure 2) to characterize the peripheral blood mononuclear cells via FACS (fluorescence activated cell sorting) analysis.

### Follow-up

Follow-up procedures after completion of adjuvant treatment have to follow standard European clinical recommendations for patients with stage II and III colon cancer: every 3 months for the first 2 years and every 6 months for the next 3 years. This includes history and physical examination, serum CEA evaluation, chest X-ray (CT scan upon suspicion of lung metastases), and abdominal ultrasound or CT scan. Clinical follow-up data will be obtained for all patients, including those with stage II disease, with a minimum follow-up time of three years.

Stage IV diseases discovered at baseline FDG-PET/CT or during the surgical intervention deemed to remove the tumor, will not be followed afterwards within the study scheme.

### Tissue bank

A tissue bank will be created from pathological blood samples and residual tumor samples taken from a surgical piece, frozen or paraffin embedded, and stored, to permit future studies with genomic profiling. Both samples from stage II and stage III tumors will be obtained.

### Health economic analysis

Efficient treatment tailoring can improve the allocation of health care resources by identifying upfront the probability of patient response to a particular treatment and identifying subgroups of patients in need of other medical approaches.

A health economic analysis will evaluate the financial impact of the strategy embodied by PePiTA and intended to improve the cost effectiveness of adjuvant treatment. This evaluation will compare the investment in FDG-PET/CT testing and preoperative FOLFOX with the potential cost savings and health benefits associated with identifying patients unlikely to respond to adjuvant chemotherapy.

### Quality of life assessment

A generic quality of life assessment (EuroQoL EQ-5D) and two neuropathy questionnaires (EORTC QLQ-CIPN20 and Modified Norris Scale) will be completed by patients each time a response is assessed and upon progression of disease. The quality of life data will be used to calculate quality-adjusted life expectancy for the trial population.

### Statistical considerations

Using data published by O' Connell and Laurie in patients with stage II-III colon cancer and a fixed-effects meta-analytic method, we determined a hazard ratio (HR) for DFS of 0.63 in favour of adjuvant 5-FU-based chemotherapy compared to follow-up only [48,49]. Because there is no evidence that the benefit of chemotherapy is different in stage II than in stage III colon cancer (Andre et al. did not identify any interaction between treatment and stage) [4], we hypothesised that this HR is applicable in a population of patients with stage III disease only. We further assumed that, for patients not responding to chemotherapy, the HR equals 1 (no benefit and no harm from chemotherapy).

We derived DFS at 3 years for stage III colon cancer from the MOSAIC trial (69%) [4]. From the previous assumptions, we expect DFS at 3 years to be 55% for non-responders and 83% for responders, in case of a 50% response rate. To detect this anticipated difference, if true, we need 33 events (α = 5% and β = 10%). To observe this number of events, we calculated that we needed 135 patients with pathological stage III disease and 225 patients to be registered in total, hypothesising that 60% of patients included in the trial will be documented with stage III colon cancer after surgery.

The trial will be regularly monitored to assess whether the observations are compatible with the expectations of 50% of eligible patients being assessed as PET responders after 1 course of FOLFOX and of 60% of screened patients with stage III. No data analysis about time-to-events variables will be conducted to make this adjustment.

## Discussion

PePiTA is the first study to use the primitive tumor's chemosensitivity assessed by serial FDG-PET/CT before and after one course of properative chemotherapy as a guidance for adjuvant therapy in colon cancer.

If its hypothesis could be demonstrated, this could pave the way for tailoring the treatment to the patients, avoiding useless toxicities and inadequate expenses for the society.

This could also give an interesting insight into tumoral heterogeneity, resistance to chemotherapy, genetic predisposants to oxaliplatin toxicity and immune response to cancer.

The study limitations may be that no fresh biopsy can be obtained from the patients before initiating therapy, due to ethical reasons impairing a redo of the diagnostic colonoscopy, and that therefore genomic analysis will be made on tumors already exposed to one course of preoperative chemotherapy, which carries the risk of a clonal selection.

### Ethical considerations

#### Patient protection

The principal investigator ensures that this study conforms to the Declaration of Helsinki (available at http://www.wma.net/en/30publications/10policies/b3/) or the laws and regulations of the country, whichever provides the greatest protection of the patient.

The study follows the International Conference on Harmonization E 6 (R1) Guideline for Good Clinical Practice, reference number CPMP/ICH/135/95 (available at http://www.emea.europa.eu/pdfs/human/ich/013595en.pdf).

The competent ethics committees of all participating centres approved the protocol, as required by applicable national legislation.

#### Trial sponsorship and financing

A Belgian National Cancer Plan grant (Belgian Group for Digestive Oncology) to the Institut Jules Bordet has provided funding for the study. The sponsor is Institut Jules Bordet – Centre des Tumeurs de l’Université Libre De Bruxelles.

## Abbreviations

CEA: Carcinoembryonic antigen; CI: Confidence interval; CRC: Colorectal carcinoma; CT: Computed tomography; CTC: Circulating tumor cells; DFS: Disease free survival; DNA: Deoxyribonucleic acid; EDTA: Ethylene diamine tetraacetic acid; EGFR: Epidermal growth factor receptor; EORTC: European Organization for Research and Treatment of Cancer; FACS: Fluorescence activated cell sorting; FDG: F18-fluorodeoxyglucose; FOLFOX-4: 5-fluorouracil, leucovorin and oxaliplatin; 5-FU: 5-fluorouracil; HR: Hazard ratio; LV: Leucovorin (folinic acid); NCI-CTCAE: National Cancer Institute – Common Toxicity Criteria for Adverse Events; MRI: Magnetic resonance imaging; NGS: Next generation sequencing; OS: Overall survival; RECIST: Response Evaluation Criteria In Solid Tumors; SNP: Single nucleotide polymorphism; SUV: Standardized uptake value; TIL: Tumor infiltrating lymphocyte; ULN: Upper limit of normal; WHO: World Health Organization.

## Competing interests

The authors declare that they have no competing interest.

## Authors’ contributions

AH, PM, VG Contribution to protocol writing, Manuscript design, Setting-up the trial, Writing manuscript; HEM, AD, CV, MM, Writing manuscript; CG, PF, Contribution to protocol writing, Manuscript design, Setting-up the trial, Writing manuscript, Coordination of PET imaging network; PM, AA, MP, Contribution to protocol writing, Setting-up the trial; JVL, Contribution to protocol writing, Setting-up the trial, Writing manuscript. All authors read and approved the final manuscript.

## Pre-publication history

The pre-publication history for this paper can be accessed here:

http://www.biomedcentral.com/1471-2407/13/190/prepub
