# Erratum: Preoperative chemosensitivity testing as Predictor of Treatment benefit in Adjuvant stage III colon cancer (PePiTA): Protocol of a prospective BGDO (Belgian Group for Digestive Oncology) multicentric study

**DOI:** 10.1186/s12885-015-1181-5

**Published:** 2015-03-25

**Authors:** Alain Hendlisz, Vassilis Golfinopoulos, Amelie Deleporte, Marianne Paesmans, Hazem El Mansy, Camilo Garcia, Marc Peeters, Lieven Annemans, Caroline Vandeputte, Marion Maetens, Marc Van den Eynde, Raphaël Maréchal, Ivan Borbath, Damien Dresse, Ghislain Houbiers, Michael Fried, Ahmad Awada, Martine Piccart, Jean-Luc Van Laethem, Patrick Flamen

**Affiliations:** 1 Medicine Department, Institut Jules Bordet, Université Libre de Bruxelles, Brussels, Belgium; 2 Data Center, Institut Jules Bordet, Université Libre de Bruxelles, Brussels, Belgium; 3 Nuclear Medicine Department, Institut Jules Bordet, Université Libre de Bruxelles, Brussels, Belgium; 4 Department of Oncology, Antwerp University Hospital, Antwerp, Belgium; 5 Health Economics, Department of Public Health, Ghent University, Ghent, Belgium; 6 Gastroenterology Department, Cliniques Universitaires Saint-Luc, Université Catholique de Louvain, Brussels, Belgium; 7 Service de Chirurgie Abdominale et Générale, Centre Hospitalier Régional La Citadelle, Liège, Belgium; 8 Service d’Oncologie et d’Hématologie, CH Saint Joseph, Liège, Belgium; 9 Oncology department, Ziekenhuis Netwerk Antwerpen, Antwerpen, Belgium; 10 Gastroenterology Department, Erasme Hospital, Université Libre de Bruxelles, Brussels, Belgium

## Erratum

After publication of this work [1], we noted that we inadvertently failed to include the complete list of all co-authors. The full list of authors and the corrected Authors Contributions section are reported below. We apologize for any inconvenience this oversight may have caused.

### Corrected Authors’ List

Alain Hendlisz^1*^, Vassilis Golfinopoulos^1^, Amelie Deleporte^1^, Marianne Paesmans^2^, Hazem El Mansy^1^, Camilo Garcia^3^, Marc Peeters^4^, Lieven Annemans^5^, Caroline Vandeputte^1^, Marion Maetens^1^, Marc Van den Eynde^6^, Raphaël Maréchal^10^, Ivan Borbath^6^, Damien Dresse^7^, Ghislain Houbiers^8^, Michael Fried^9^, Ahmad Awada^1^, Martine Piccart^1^, Jean-Luc Van Laethem^10^ and Patrick Flamen^3^

### Corrected Authors' contributions

AH, PM, VG Contribution to protocol writing, Manuscript design, Setting-up the trial, Writing manuscript; HEM, AD, CV, MM, MVDE, RM, Writing manuscript; CG, PF, Contribution to protocol writing, Manuscript design, Setting-up the trial, Writing manuscript, Coordination of PET imaging network; PM, AA, MP, Contribution to protocol writing, Setting-up the trial; JVL, Contribution to protocol writing, Setting-up the trial, Writing manuscript. All authors read and approved the final manuscript.
